# Research on mix design and mechanical performances of MK-GGBFS based geopolymer pastes using central composite design method

**DOI:** 10.1038/s41598-024-59872-0

**Published:** 2024-04-20

**Authors:** Ziqi Yao, Ling Luo, Yongjun Qin, Jiangbo Cheng, Changwei Qu

**Affiliations:** 1 College of Civil Engineering and Architecture, Xinjiang University, Urumqi, 830017 China; 2 Xinjiang Civil Engineering Technology Research Center, Urumqi, 830017 China

**Keywords:** Metakaolin, Slag, Central composite design, Workability, Compressive strength, Civil engineering, Structural materials, Mechanical properties

## Abstract

In order to alleviate environmental problems and reduce CO_2_ emissions, geopolymers had drew attention as a kind of alkali-activated materials. Geopolymers are easier access to raw materials, green and environment friendly than traditional cement industry. Its special reaction mechanism and gel structure show excellent characteristics such as quick hardening, high strength, acid and alkali resistance. In this paper, geopolymer pastes were made with metakaolin (MK) and ground granulated blast furnace slag (GGBFS) as precursors. The effects of liquid–solid ratio (L/S) and modulus of sodium silicate (Ms) on the performances of MK-GGBFS based geopolymer paste (MSGP) were characterized by workability, strength and microstructural tests. The regression equations were obtained by central composite design method to optimize the mix design of MSGP. The goodness of fit of all the equations were more than 98%. Based on the results of experiments, the optimum mix design was found to have L/S of 0.75 and Ms of 1.55. The workability of MSGP was significantly improved while maintaining the strength under the optimum mix design. The initial setting time of MSGP decreased by 71.8%, while both of the fluidity and 28-d compressive strength increased by 15.3%, compared with ordinary Portland cement pastes. Therefore, geopolymers are promising alternative cementitious material, which can consume a large amount of MK and GGBFS and promote green and clean production.

## Introduction

Ground granulated blast furnace slag (GGBFS) is a solid waste produced from blast furnaces during pig iron smelting. About 0.3–1.0 t of blast furnace slag is produced for every 1 t of iron produced. In China, the production of industrial solid waste was as high as 3.787 billion t in 2020, of which 0.69 billion t of metallurgical waste slag accounting for 18.19%^1^. Meanwhile, the generation of industrial solid waste is accompanied with the emission of greenhouse gases. It is estimated that the year-on-year growth of CO_2_ emissions rose from 0.9% in the 1990s to 3% in the 2000s, while annual emissions of CO_2_ are nearly 29.6 billion t and on an increasing trend^2,3^. The traditional cement industry accounts for about 8–9% of total anthropogenic CO_2_ emissions^4^. To mitigate the situation, GGBFS can be considered as a raw material to produce geopolymers. Geopolymer is a kind of alkali-activated materials, which are typically made from GGBFS, metakaolin (MK), and fly ash (FA)^5–7^. These materials have excellent properties such as high compressive strength, good durability, high temperature resistance, and well acid resistance^8–11^. Compared with the production of ordinary Portland cement (OPC), the CO_2_ emissions of alkali-activated materials can be reduced by up to 80%, ensuring material performance and achieving the aim of green and energy-saving at the same time^12^.

Several attempts in different aspects have been made to broaden the application of geopolymers in the construction industry^13–17^. Based on previous research, GGBFS-based alkali-activated materials tends to have poor workability, high drying shrinkage and quick setting, while MK-based alkali-activated materials are characterized by slow setting and mitigation of the drying shrinkage^18–23^. A good synergy of GGBFS and MK in alkali-activated materials could obtain both good workability, mechanical strength and durability^23–25^. The study of Alanazi et al. pointed out that, the partial replacement of FA with MK significantly enhanced the early strength (the strength at 3 days increased from 14 to 30 MPa)^26^. Zhang et al. proved that under normal temperature curing conditions, the mechanical performance of MK-based geopolymers were similar to those of OPC, and often exhibits higher flexural strength^27^. Habert et al. addressed that geopolymer concrete made from FA/GGBFS require less sodium silicate solution to activate^28^. Therefore, they have a lower environmental impact than geopolymer concrete made from pure MK.

Besides, the mix design of different precursors based geopolymer is complex, including many influencing factors, as modulus of sodium silicate (Ms), liquid–solid ratio (L/S), curing conditions, and others^29–33^. Danish et al. studied the effects of Ms and curing conditions on the properties of prepacked geopolymer mixes. The findings suggested that the specimens cured for 8 h with a given Ms performed higher compressive and flexural strength^29^. Zhang et al. found that the strongest unconfined compressive strength (UCS) of solid alkali-activated geopolymers was obtained when L/S and Ms were 0.64 and 1.16^34^. According to the research of Wang et al., the performance of FA-based geopolymer paste was effectively improved while maintaining the strength by increasing the alkali-activator, decreasing Ms, and adjusting the water-binder ratio (w/b)^35^.

Response surface methodology is a collection of statistical and mathematical techniques, which achieves its goal by improving the settings of the factors, bringing the response closer and closer to the predetermined maximum or minimum value^36–38^. Response surface methodology can be used in the process of designing, developing, and building new products, as well as in improving existing products designs^39–43^. Meanwhile, the number of trials can be minimized by response surface methodology and identify the interactions between factors at-a-time^44^. Central composite design (CCD) is the most popular response surface methodology in use, in which the axial distance and the number of center runs can be flexibly selected^37,45^. Response surface methodology uses the factorial methods and Analysis of Variance (ANOVA) to model the response values. On top of this, CCD adds extra factors, both within (at the focal point) and outside of the factor region (at the star point), to highlight the results and enhance the predictive capacity of the models^46^.

Many scholars used CCD to optimize the experiment process and distinguish the interactions between factors. Watson et al. identified the interactions between As and natural organic matter during the ferric chloride coagulation via CCD^44^. In order to maximum the properties of the electrospun nanofiber, Rooholghodos et al. used CCD to optimized the crosslinking duration and CQDs-Fe_3_O_4_-RE concentration^47^. Du et al. found the optimum mix proportion of high-volume FA mortar using CCD^48^. However, the studies of improving the performances of geopolymer pastes using CCD were limited.

In the present study, the MK-GGBFS binary composite system was used as the precursor to produce geopolymer paste, and L/S and Ms were selected as the experimental variables. The models of fluidity, initial setting time and UCS (3-d, 7-d, 28-d, 60-d) were established by CCD method. Then, the microstructure was characterized by SEM and XRD. Ultimately, the optimum mix design of MSGP was employed to ensure the workability and mechanical performance.

## Materials and experimentation

### Materials

The Blaine fineness of MK and GGBFS were 620 and 430 m^2^/kg, respectively. The basicity coefficient K_b_ = (CaO + MgO)/(SiO_2_ + Al_2_O_3_) of GGBFS was 1.23. The OPC was Tianshan P·O 42.5R cement. The chemical compositions of these materials are listed in Table 1. The alkali-activator was prepared by mixing NaOH particles (96%) and sodium silicate solution in a certain proportion. The chemical composition of sodium silicate solution was SiO_2_ (26.6%) and Na_2_O (8.7%), the original modulus was 3.16. In the trial test, the activator concentration of 36%, 37%, 38%, 39% and 40% were used, and it was easier to mix at 37%. Therefore the activator concentration was set at 37% in this study.

**Table 1:** Main chemical composition of raw materials (wt%).

Materials	CaO	SiO_2_	Al_2_O_3_	Fe_2_O_3_	SO_3_	MgO	K_2_O	Na_2_O	LOI
MK	0.65	51.59	40.05	2.30	0.41	2.17	1.57	0.83	1.88
GGBFS	43.15	29.20	12.59	1.44	2.00	8.09	0.45	0.52	0.73
Cement	56.78	25.52	7.51	2.89	2.43	1.33	0.67	0.49	1.45

**Figure 1:**
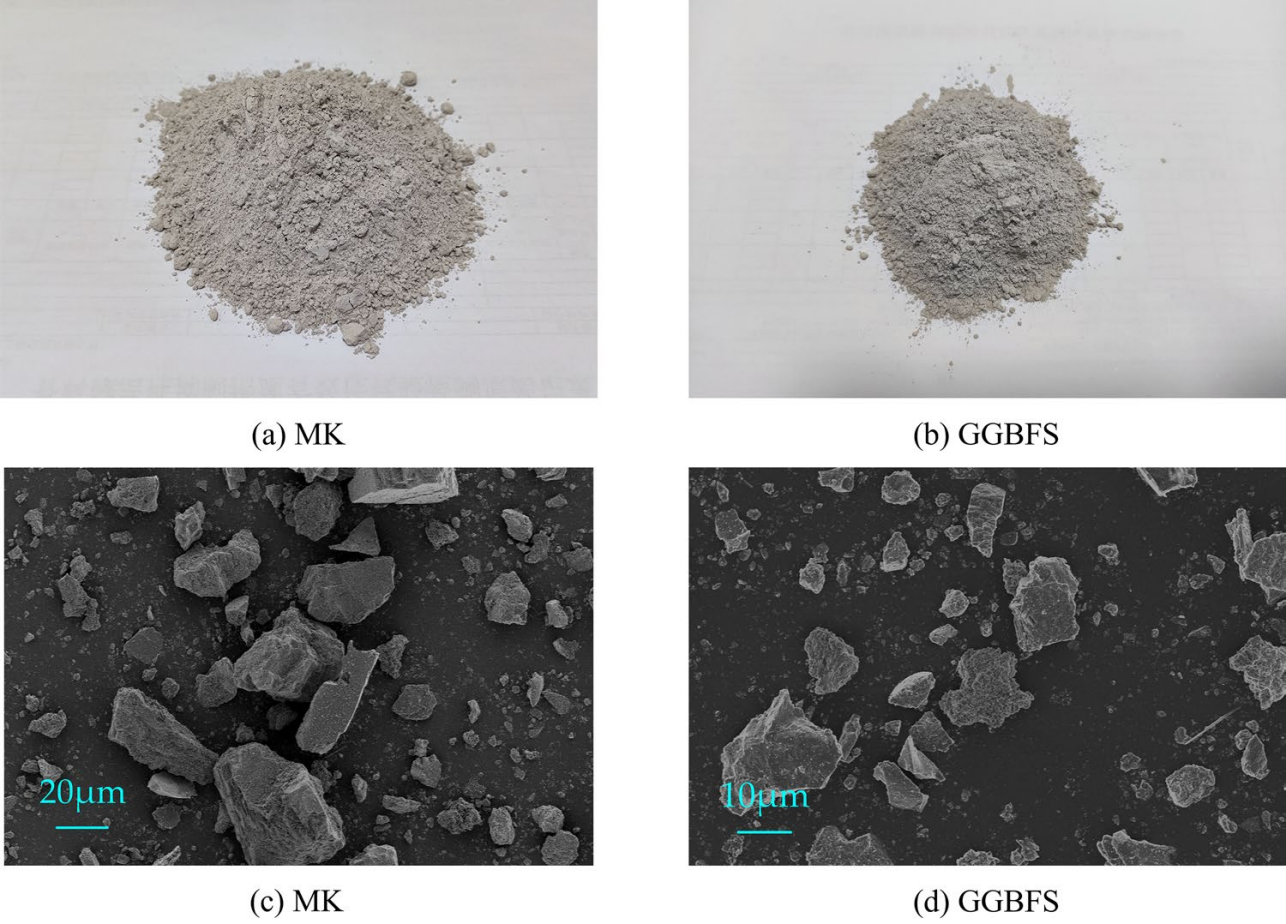
Physical photo and SEM of MK and GGBFS.

**Figure 2:**
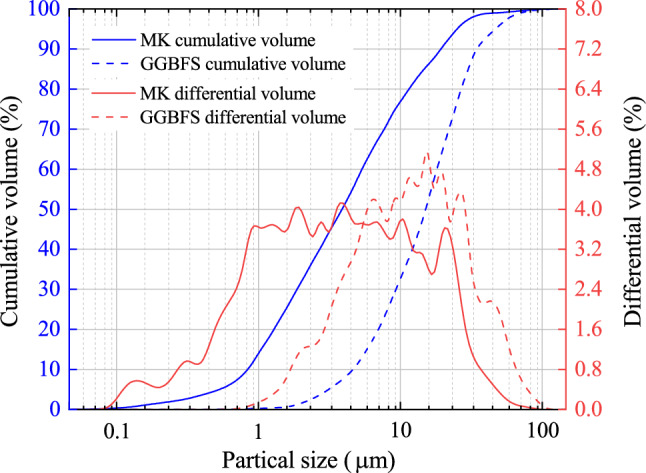
Particle size curves of MK and GGBFS.

**Figure 3:**
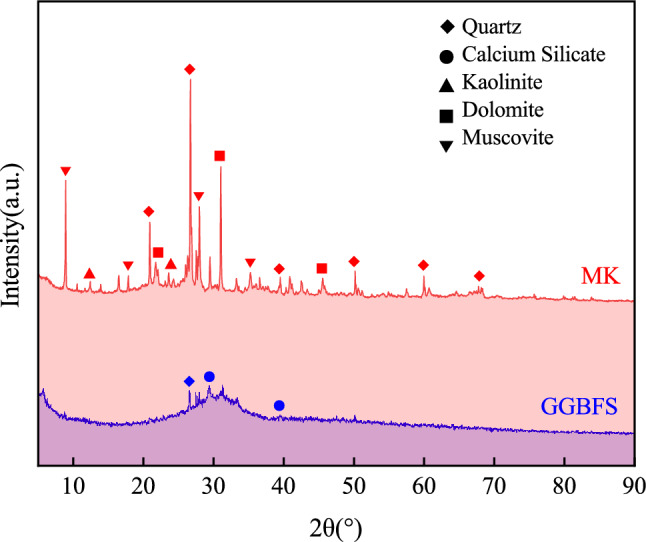
XRD patterns of MK and GGBFS.

The microstructure of MK and GGBFS samples were characterized by Sigma-300 SEM produced by ZEISS, which was illustrated in Fig. 1. Mastersizer-2000 Laser diffraction tester produced by MALVERN examined the particle size distribution of MK and GGBFS, which were shown in Fig. 2. The D50 (average particle size) of GGBFS and FA are about 4.52 μm and 18.6 μm. Figure 3 presents the XRD patterns of MK and GGBFS. It is obvious that MK includes many crystalline phases such as quartz (SiO_2_), kaolinite (Al_4_[Si_4_O_10_](OH)_8_), calcium silicate (C_2_S and C_3_S), dolomite (CaMg(CO_3_)_2_) and muscovite (K{Al_2_[AlSi_3_O_10_](OH)_2_}). The humps centring at the 2θ range of 20–30° of MK and 25–35° of GGBFS reflect an amorphous phase^49,50^.

### Mix design of MSGP based on CCD method

In this study, CCD method was used to investigate the effect of L/S and Ms on the performance of MSGP. According to the relevant studies and a trail test, the primary variation ranges of L/S and Ms were determined^31,34,51–54^. L/S ratios were set at 0.7, 0.8, 0.9 and 1.0, and Ms values were set at 1.2, 1.5 and 1.8. The total amount of binders were 450 g. MK and GGBFS were equal as 225 g. The control group OPC pastes had a w/b of 0.5, which using cement as binders and no admixtures. The response values are fluidity, initial setting time, and UCS (3-d, 7-d, 28-d, 60-d). Table 2 summarizes the factors, codes and levels of MSGP mix design under the CCD method.

**Table 2:** Codes and levels of factors for CCD.

Factors	Code	Level				
		− 1.414	− 1	0	1	1.414
L/S	*x*_*1*_	0.66	0.7	0.8	0.9	0.94
Ms	*x*_*2*_	1.08	1.2	1.5	1.8	1.92

### Experimental methods

#### Setting time

MSGP was prepared according to the mix design of the CCD method. The initial and final setting times of pastes were measured referred to Chinese Standard GB/T 1346–2011^55^. The samples used to test the setting time of OPC pastes should be standard consistency ones.

#### Fluidity

The fluidity tests of freshly mixed MSGP pastes were conducted according to Chinese Standard GB/T 8077–2012^56^. The maximum diameter in two directions perpendicular to each other were measured by calipers, and the average value was taken as fluidity.

#### Compressive strength

The compressive strength on 40 × 40 × 40 mm hardened paste cubes was tested per Chinese Standard GB/T 17,671–2021, which was the average of 3 samples for each group^57^.

#### SEM and XRD

The microstructure and hydration products of samples were characterized by SEM and XRD, respectively. All samples to be measured were soaked in absolute ethanol immediately for 72 h to stop hydration, and placed in an electric thermostatic drying oven for drying. The powdered samples used in XRD were ground after drying and passed through a sieve of 0.075 mm, then packed for testing. The parameters of the XRD were as follows: copper target, 30 kV, 5–90°, 5°/min.

### Experimental results analysis

#### Influence of L/S and Ms on workability

**Figure 4:**
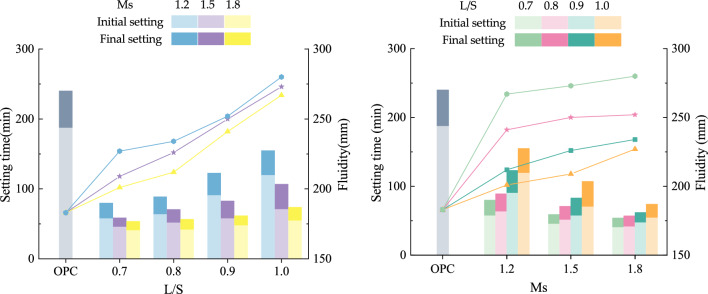
Influence of L/S and Ms on workability.

The effects of L/S and Ms were clarified on setting time and fluidity in Fig. 4. It was obvious that the fluidity was significantly improved as L/S increased, while the impact of Ms on fluidity was not such clear. For the setting time, it kept an increasing trend with the increase of L/S. In contrast, when Ms increased, the setting time became shorter. Obviously, all MSGP had higher fluidity than OPC pastes.

Since the activator concentration was 37%, MSGP with L/S of 0.8 had the same water content as OPC pastes with w/b of 0.5. For comparison, when L/S = 0.8, Ms = 1.2, 1.5, 1.8, the fluidities were 212, 226, and 234 mm, respectively, which were 15.8%, 23.5%, and 27.9% higher than that of OPC pastes (183 mm). This was because sodium silicate acted not only as a solvent, but also as a gel. From the perspective of microstructure, it could be considered that sodium silicate was a dispersion of amorphous silicate colloids in an alkaline aqueous medium^58^. Therefore, the interaction between MK and GGBFS particles weakened and improved the fluidity of the fresh pastes. For MK, its tabular granular and clay structure led to higher water demand. The incorporation of GGBFS reduced the amount of MK, so it improved the fluidity of MSGP when the water consumption was fixed. With the increase of Ms, the fluidity of MSGP increased, and the fluidity of Ms = 1.8 was increased by 4.6–12.9% compared to Ms = 1.2 under the same L/S. The reason was the formation of independent silicate micelles in solution at high modulus (< 2.5) helped to disperse precursors particles and improve the rheological properties of the pastes^59^.

On the other hand, it was noted that L/S and Ms had an opposite effect on the setting time. In Fig. 4, as L/S increased, the setting time of MSGP prolonged. The time required for the paste to lose fluidity was related to its kinetics. The concentration of active ingredients in the dissolved medium decreased when the water content was high, hence the time required to convert free water to bound water increased accordingly^5^. However, the increase of Ms played an accelerated role in setting. When Ms was 1.2, the initial setting time of MSGP was about 58–120 min, while the initial setting time of MSGP was greatly shortened to 41–55 min after Ms was increased to 1.8. Therefore, the high Ms would shorten the setting time of MSGP. The reason for the shortened setting time is mainly related to Ca^2+^ as charge-balanced ions^60,61^. Ca^2+^ has a stronger charge attraction and neutralization, so the formation of aluminosilicate gels will be faster. At the same time, the presence of Ca^2+^ will cause heterogeneous nucleation effect in the initial reaction process of geopolymers^62^. Heterogeneous nucleation effect also accelerate the formation of geopolymer gels, resulting in a shorter setting time.

#### Influence of L/S and Ms on UCS and mass loss

**Figure 5:**
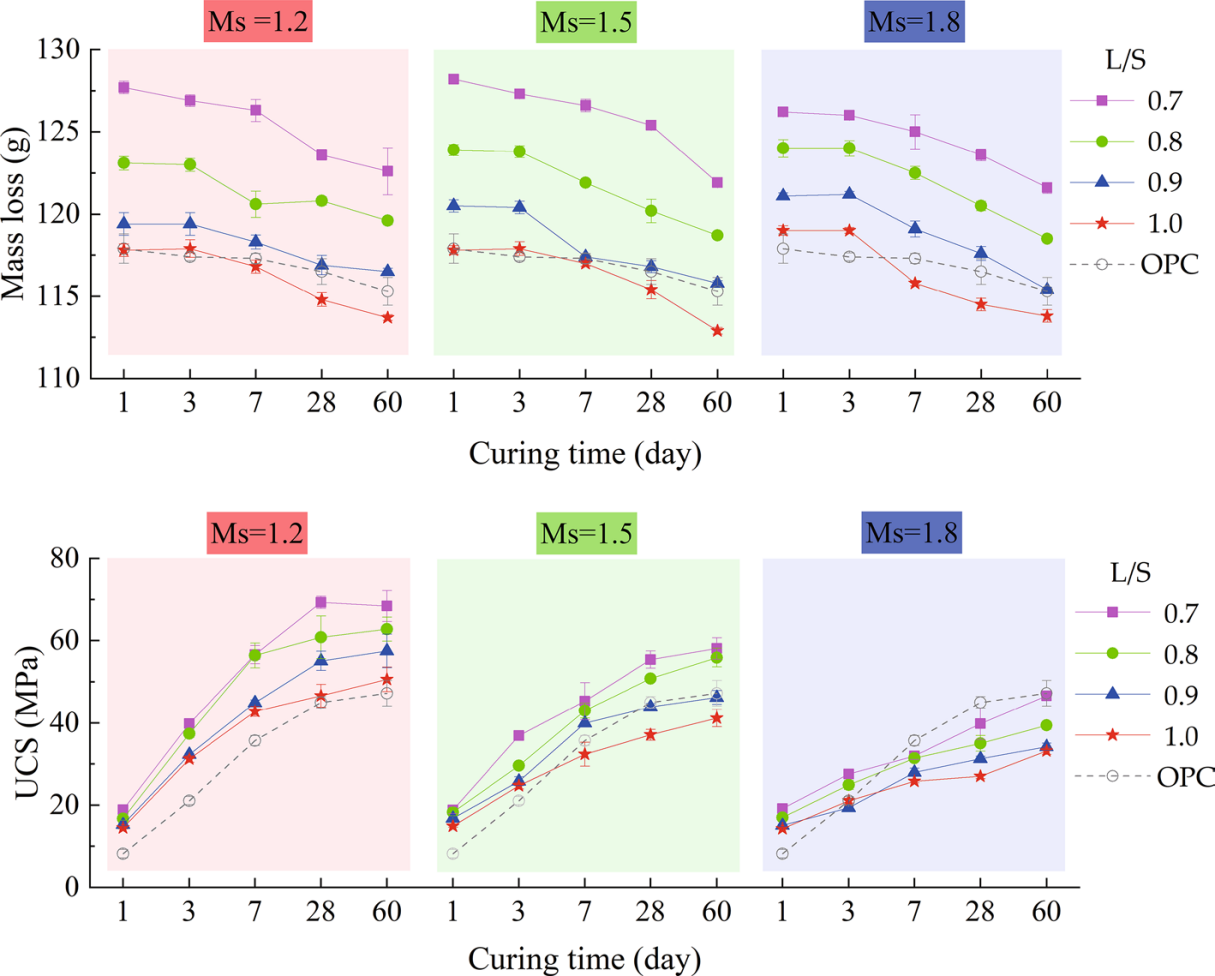
Influence of Ms and L/S on UCS and mass loss.

It was illustrated in Fig. 5 that the comparison of UCS and mass loss changes of each group under different L/S and Ms. The experimental results found that when Ms was constant, the UCS of MSGP decreased with the increase of L/S. When Ms was 1.2, with L/S increased from 0.7 to 1.0, the 28-d UCS were 69.3, 60.8, 59.5, and 46.6 MPa, which were higher than OPC pastes (45.0 MPa). While they were 36.3, 32.0, 31.3, and 27.0 MPa when Ms was 1.8, which were lower than OPC pastes. The UCS of each group was closer to OPC pastes when Ms was 1.5. In summary, the increase of L/S was not conducive to the hardening performance of MSGP, similar to the influence of w/b on the UCS of OPC pastes. According to Davisovits and Heah et al., the fluid medium is more than the solid in the mix when L/S is high^31,63^. The contact distance between the activating solution and the precursors is far and limited because of the large volume of the fluid medium, and the dissolution of the aluminosilicate precursor is slow. Instead, when lower L/S is employed, the contact distance between the activating solution and the precursors is improved and the UCS is enhanced as a result.

However, unlike pure water, activators were usually mixed solutions consisting of alkali, soluble silicon, and water, which greatly affected the driving forces of hydration. From Fig. 5, it can be found that when Ms increased from 1.2 to 1.5, the UCS of each group decreased significantly. When L/S was 0.8, The 28-d UCS of the three groups was 60.8, 50.8, and 32.0 MPa, respectively. Especially in the process of Ms increased from 1.5 to 1.8, the UCS decreased more obviously. It was observed that the variation of the UCS (curing time > 28 day) of the three groups of MSGP with different Ms was various. When Ms was 1.2, the 60-d UCS was 68.4, 62.8, 60.6, and 53.2 MPa, which decreased by 1.3% and increased by 3.3%, 1.8%, and 14.2% compared with the 28-d UCS respectively. The increase of UCS was not obvious. While Ms was 1.5, the 60-d UCS was 60.1, 54.2, 51.2, and 44.2 MPa, which increased by 8.5%, 6.7%, 16.6%, and 19.1%. The 60-d UCS increased most when Ms increased to 1.8, which was 22.9%, 23.1%, 14.1%, and 23.0%, respectively, reaching 44.6, 39.4, 35.7, and 33.2 MPa.

It is not difficult to see that the high Ms played a more critical role in the late-age strength development, which was due to the difference in the composition of the activators. For the main components of the modified activator (Na_2_SiO_3_, NaOH), NaOH provides higher solution alkalinity, and the solubility of aluminosilicate is greater under strong alkaline environment, which promotes the polymerization reaction and improves the mechanical properties. When preparing high-modulus sodium silicate solution, it requires less NaOH and leads lower Na_2_O content, which inhibits the interactions between active substances and weakening the development of mechanical properties^64^. For the MK-GGBFS system, the high reactivity of GGBFS improves the early reaction of MK-based geopolymers. Increasing Ms within a certain range (it is pointed out that Ms < 2.0^65^) can improve the strength development at 28 days or longer.

## Results and discussion

### Results of CCD method

A total of 13 random mix design tests were performed (include 5 center-point repeat tests) based on the CCD method of the Design-expert software. The mix design and responses are shown in Table 3. The code for the factor 1 L/S is *x*_*1*_, the code for the factor 2 Ms is *x*_*2*_. Response 1 is fluidity (mm), response 2 is initial setting time (min), and response 3, 4, 5, 6 is 3-d, 7-d, 28-d, and 60-d UCS (MPa), respectively.

**Table 3:** CCD scheme and experimental results.

No	*x*_*1*_	*x*_*2*_	Fluidity (mm)	Initial setting time (min)	UCS (MPa)			
					3-d	7-d	28-d	60-d
1	0.9	1.2	241	91	32.3	44.9	55.1	57.5
2	0.8	1.5	226	52	29.6	43.0	50.8	54.2
3	0.8	1.5	221	59	31.0	41.7	50.3	54.7
4	0.8	1.07	204	69	43.3	57.9	62.1	64.5
5	0.94	1.5	263	71	24.9	33.7	39.1	45.8
6	0.7	1.8	227	41	27.6	32.0	39.8	46.6
7	0.65	1.5	198	43	43.5	48.1	56.9	62.2
8	0.8	1.5	229	54	29.9	43.5	50.3	54.5
9	0.8	1.92	241	38	23.5	30.4	31.1	38.6
10	0.9	1.8	252	48	19.4	28.0	31.3	34.2
11	0.8	1.5	227	57	32.5	43.9	53.1	56.3
12	0.8	1.5	225	51	29.3	42.9	51.1	54.4
13	0.7	1.2	201	58	39.8	56.6	69.3	68.4

### Response surface model fitting and verification

Regression fitting analysis was conducted with the experimental data in Table 4. The fitting functions are shown as follows:

1 $$\begin{aligned} {\text{Fluidity }} & { = 225}{\text{.6 + 22}}{\text{.98x}}_{{1}} { + 13}{\text{.08x}}_{{2}} - { 3}{\text{.75x}}_{{1}} {\text{x}}_{{2}} { + 2}{\text{.45x}}_{{1}}^{{2}} \\ & \quad - {1}{\text{.55x}}_{2}^{2} - {3}{\text{.83x}}_{1}^{2} x_{2} { } - {6}{\text{.73x}}_{1} x_{2}^{2} { + 3}{\text{.75x}}_{1}^{2} x_{2}^{2} \\ \end{aligned}$$

2 $$\begin{aligned} {\text{Initial }}\;{\text{setting}}\;{\text{ time }} & { = 54}{\text{.6 + 9}}{\text{.9x}}_{{1}} { } - { 10}{\text{.96x}}_{{2}} { } - { 6}{\text{.5x}}_{{1}} {\text{x}}_{{2}} { + 1}{\text{.20x}}_{{1}}^{{2}} \\ & \quad - { 0}{\text{.55x}}_{2}^{2} - {4}{\text{.04x}}_{1}^{2} x_{2} { + 0}{\text{.1005x}}_{1} x_{2}^{2} { + 4}{\text{.25x}}_{1}^{2} x_{2}^{2} \\ \end{aligned}$$

3 $$\begin{aligned} {\text{3 - d UCS }} & { = 30}{\text{.46 }} - { 6}{\text{.58x}}_{{1}} - {\text{ 7x}}_{{2}} { } - {0}{\text{.175x}}_{{1}} {\text{x}}_{{2}} { + 1}{\text{.87x}}_{{1}}^{{2}} \\ & \quad { + 1}{\text{.47x}}_{{2}}^{{2}} { + 0}{\text{.7254x}}_{{1}}^{{2}} {\text{x}}_{{2}} { + 2}{\text{.65x}}_{{1}} {\text{x}}_{{2}}^{{2}} { } - {4}{\text{.02x}}_{{1}}^{{2}} {\text{x}}_{{2}}^{{2}} \\ \end{aligned}$$

4 $$\begin{aligned} {\text{7 - d UCS }} & { = 43 } - { 5}{\text{.09x}}_{{1}} { } - {9}{\text{.72x}}_{{2}} { + 1}{\text{.93x}}_{{1}} {\text{x}}_{{2}} { - 1}{\text{.05x}}_{{1}}^{{2}} { + 0}{\text{.575x}}_{{2}}^{{2}} \\ & \quad - { 0}{\text{.6523x}}_{1}^{2} x_{2} { + 1}{\text{.17x}}_{1} x_{2}^{2} { } - {2}{\text{.15x}}_{1}^{2} x_{2}^{2} \\ \end{aligned}$$

5 $$\begin{aligned} {\text{28 - d UCS }} & { = 51}{\text{.12}} - { 6}{\text{.29x}}_{1} { } - { 10}{\text{.96x}}_{2} { + 1}{\text{.43x}}_{1} x_{2} - { 1}{\text{.56x}}_{1}^{2} \\ & \quad - { 2}{\text{.26x}}_{2}^{2} { } - {2}{\text{.36x}}_{1}^{2} x_{2} { + 0}{\text{.6183x}}_{1} x_{2}^{2} { + 1}{\text{.57x}}_{1}^{2} x_{2}^{2} \\ \end{aligned}$$

6 $$\begin{aligned} {\text{60 - d UCS }} & { = 54}{\text{.82 }} - {5}{\text{.8x}}_{1} { } - { 9}{\text{.16x}}_{2} { } - { 0}{\text{.375x}}_{1} x_{2} { } - {0}{\text{.41x}}_{1}^{2} \\ & \quad - { 1}{\text{.63x}}_{2}^{2} { } - { 2}{\text{.12x}}_{1}^{2} x_{2} - {0}{\text{.0267x}}_{1} x_{2}^{2} - { 1}{\text{.1x}}_{1}^{2} x_{2}^{2} \\ \end{aligned}$$

**Table 4:** Model validation for the responses.

Response	Fluidity	Initial setting time	3-d UCS	7-d UCS	28-d UCS	60-d UCS
Standard deviation	2.97	3.36	1.31	0.8307	1.16	0.8468
C.V.(%)	1.31	5.97	4.18	1.98	2.35	1.59
R^2^	0.9920	0.9816	0.9892	0.9973	0.9965	0.9975
Adj. R^2^	0.9760	0.9449	0.9676	0.9919	0.9895	0.9925
Adeq precision	26.3343	18.9490	22.1304	43.2611	39.6312	48.5419
F-value	62.04	26.70	45.73	184.06	141.70	199.41
*p*-value	0.0006	0.0033	0.0011	< 0.0001	0.0001	< 0.0001
Significance	Yes	Yes	Yes	Yes	Yes	Yes

Model validation was performed on the above response surface functions, and the results are shown in Table 4.

**Figure 6:**
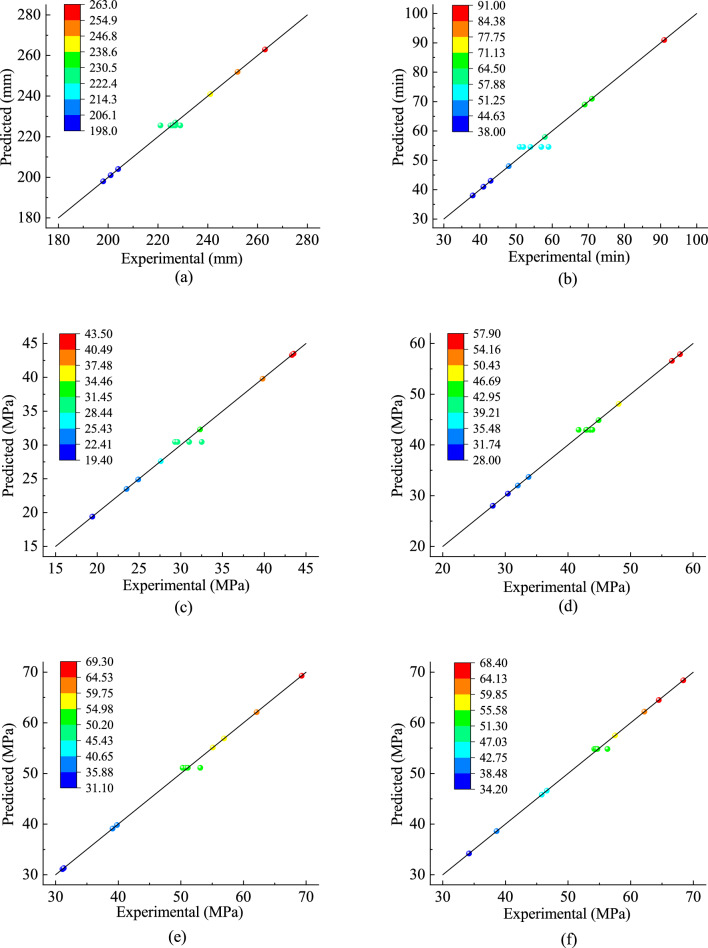
Comparison of predicted and experimental values:(**a**) Fluidity; (**b**) Initial setting time; (**c**) 3-d UCS; (**d**) 7-d UCS; (**e**) 28-d UCS; and (**f**) 60-d UCS.

Table 4 showed that the *p*-values of the regression models of the fluidity, initial setting time, and UCS (3-d, 7-d, 28-d, 60-d) were all < 0.01, indicating that these six mathematical models were statistically significant. The R^2^ of the fitting equations were 0.9920, 0.9816, 0.9892, 0.9973, 0.9965, and 0.9975, respectively, which indicated that the six statistical models could explain the changes in response values of 99.20%, 98.16%, 98.92%, 99.73%, 99.65%, and 99.75%. It informed that the predicted values agree with the actual results approximately and the experimental error was not obvious. In addition, the C.V. of the models were all less than 10%, which showed that the experiment had high reliability and precision. The adequate precision greater than 4 could be considered as desirable, and all the equations above are satisfied. Figure 6 illustrates the relationships between the predicted values and the experimental values.

### ANOVA and interaction

The ANOVA of the models of the fluidity, initial setting time, UCS (3-d, 7-d, 28-d, 60-d) are shown in Tables 5–7. From the statistical hypothesis testing, if the *p*-value ≤ 0.05, this factor is considered to have a significant effect on the response value, and vice versa^66^. The *p*-values of the above six regression equations were all less than 0.01, it could be considered that the fitting of the model is statistically significant. While the *p*-values of the lack of fit of each equation were greater than 0.05, there was a tiny discrepancy between the model and the experimental results. That was, the model fitted well. For the factor interactions under each response value, the *p*-value of each item was greater than 0.05, which had almost no effect on the response values.

**Table 5:** ANOVA of regression model.

Response	Variation Source	Sum of Squares	df	Mean Square	F-value	*p*-value
Fluidity	Model	4367.57	8	545.95	62.04	0.0006
	*x*_*1*_	2112.50	1	2112.50	240.06	0.0001
	*x*_*2*_	684.50	1	684.50	77.78	0.0009
	*x*_*1*_*x*_*2*_	56.25	1	56.25	6.39	0.0648
	*x*_*1*_^*2*^	34.30	1	34.30	3.90	0.1196
	*x*_*2*_^*2*^	13.73	1	13.73	1.56	0.2798
	*x*_*1*_^*2*^*x*_*2*_	29.36	1	29.36	3.34	0.1418
	*x*_*1*_*x*_*2*_^*2*^	90.61	1	90.61	10.30	0.0326
	*x*_*1*_^*2*^*x*_*2*_^*2*^	28.13	1	28.13	3.20	0.1483
	Residual	327.24	10	32.72		
	Lack of Fit	292.04	6	48.67	5.53	0.0599
	Pure Error	35.20	4	8.80		
	Cor Total	4402.77	12

**Table 6:** ANOVA of initial setting time regression model.

Response	Variation Source	Sum of Squares	df	Mean Square	F-value	*p*-value
Initial setting time	Model	2413.57	8	301.70	26.70	0.0033
	*x*_*1*_	392.00	1	392.00	34.69	0.0042
	*x*_*2*_	480.50	1	480.50	42.52	0.0029
	*x*_*1*_*x*_*2*_	169.00	1	169.00	14.96	0.0180
	*x*_*1*_^*2*^	8.23	1	8.23	0.7282	0.4416
	*x*_*2*_^*2*^	1.73	1	1.73	0.1530	0.7157
	*x*_*1*_^*2*^*x*_*2*_	32.64	1	32.64	2.89	0.1644
	*x*_*1*_*x*_*2*_^*2*^	0.0202	1	0.0202	0.0018	0.9683
	*x*_*1*_^*2*^*x*_*2*_^*2*^	36.13	1	36.13	3.20	0.1483
	Residual	149.93	9	16.66		
	Lack of Fit	104.73	5	20.95	1.85	0.2848
	Pure Error	45.20	4	11.30		
	Cor Total	2458.77	12

**Table 7:** ANOVA of the UCS regression model.

Response	Variation Source	Sum of Squares	df	Mean Square	F-value	*p*-value
3-d UCS	Model	626.71	8	78.34	45.73	0.0011
	*x*_*1*_	172.98	1	172.98	100.98	0.0006
	*x*_*2*_	196.02	1	196.02	114.43	0.0004
	*x*_*1*_*x*_*2*_	0.1225	1	0.1225	0.0715	0.8024
	*x*_*1*_^*2*^	19.98	1	19.98	11.67	0.0269
	*x*_*2*_^*2*^	12.35	1	12.35	7.21	0.0550
	*x*_*1*_^*2*^*x*_*2*_	1.05	1	1.05	0.6143	0.4770
	*x*_*1*_*x*_*2*_^*2*^	14.06	1	14.06	8.21	0.0457
	*x*_*1*_*x*_*2*_^*2*^	32.40	1	32.40	18.91	0.0122
	Residual	60.55	10	6.05		
	Lack of Fit	53.69	6	8.95	5.22	0.0657
	Pure Error	6.85	4	1.71		
	Cor Total	633.56	12			
7-d UCS	Model	1016.01	8	127.00	184.06	< 0.0001
	*x*_*1*_	103.68	1	103.68	150.26	0.0003
	*x*_*2*_	378.13	1	378.13	548.01	< 0.0001
	*x*_*1*_*x*_*2*_	14.82	1	14.82	21.48	0.0098
	*x*_*1*_^*2*^	6.30	1	6.30	9.13	0.0391
	*x*_*2*_^*2*^	1.89	1	1.89	2.74	0.1733
	*x*_*1*_^*2*^*x*_*2*_	0.8509	1	0.8509	1.23	0.3290
	*x*_*1*_*x*_*2*_^*2*^	2.72	1	2.72	3.94	0.1181
	*x*_*1*_^*2*^*x*_*2*_^*2*^	9.24	1	9.24	13.40	0.0216
	Residual	15.58	7	2.23		
	Lack of Fit	12.82	3	4.27	6.19	0.0553
	Pure Error	2.76	4	0.6900		
	Cor Total	1018.77	12			
28-d UCS	Model	1521.30	8	190.16	141.70	0.0001
	*x*_*1*_	158.42	1	158.42	118.05	0.0004
	*x*_*2*_	480.50	1	480.50	358.05	< 0.0001
	*x*_*1*_*x*_*2*_	8.12	1	8.12	6.05	0.0697
	*x*_*1*_^*2*^	13.91	1	13.91	10.36	0.0323
	*x*_*2*_^*2*^	29.19	1	29.19	21.75	0.0096
	*x*_*1*_^*2*^*x*_*2*_	11.18	1	11.18	8.33	0.0447
	*x*_*1*_*x*_*2*_^*2*^	0.7645	1	0.7645	0.5696	0.4924
	*x*_*1*_^*2*^*x*_*2*_^*2*^	4.96	1	4.96	3.70	0.1269
	Residual	22.28	7	3.18		
	Lack of Fit	16.91	3	5.64	4.20	0.0997
	Pure Error	5.37	4	1.34		
	Cor Total	1526.67	12			
60-d UCS	Model	1143.82	8	142.98	199.41	< 0.0001
	*x*_*1*_	134.48	1	134.48	187.56	0.0002
	*x*_*2*_	335.40	1	335.40	467.79	< 0.0001
	*x*_*1*_*x*_*2*_	0.5625	1	0.5625	0.7845	0.4258
	*x*_*1*_^*2*^	0.9606	1	0.9606	1.34	0.3115
	*x*_*2*_^*2*^	15.28	1	15.28	21.30	0.0099
	*x*_*1*_^*2*^*x*_*2*_	8.97	1	8.97	12.51	0.0241
	*x*_*1*_*x*_*2*_^*2*^	0.0014	1	0.0014	0.0020	0.9665
	*x*_*1*_^*2*^*x*_*2*_^*2*^	2.42	1	2.42	3.38	0.1401
	Residual	14.26	7	2.04		
	Lack of Fit	11.39	3	3.80	5.30	0.0705
	Pure Error	2.87	4	0.7170		
	Cor Total	1146.68	12			

**Figure 7:**
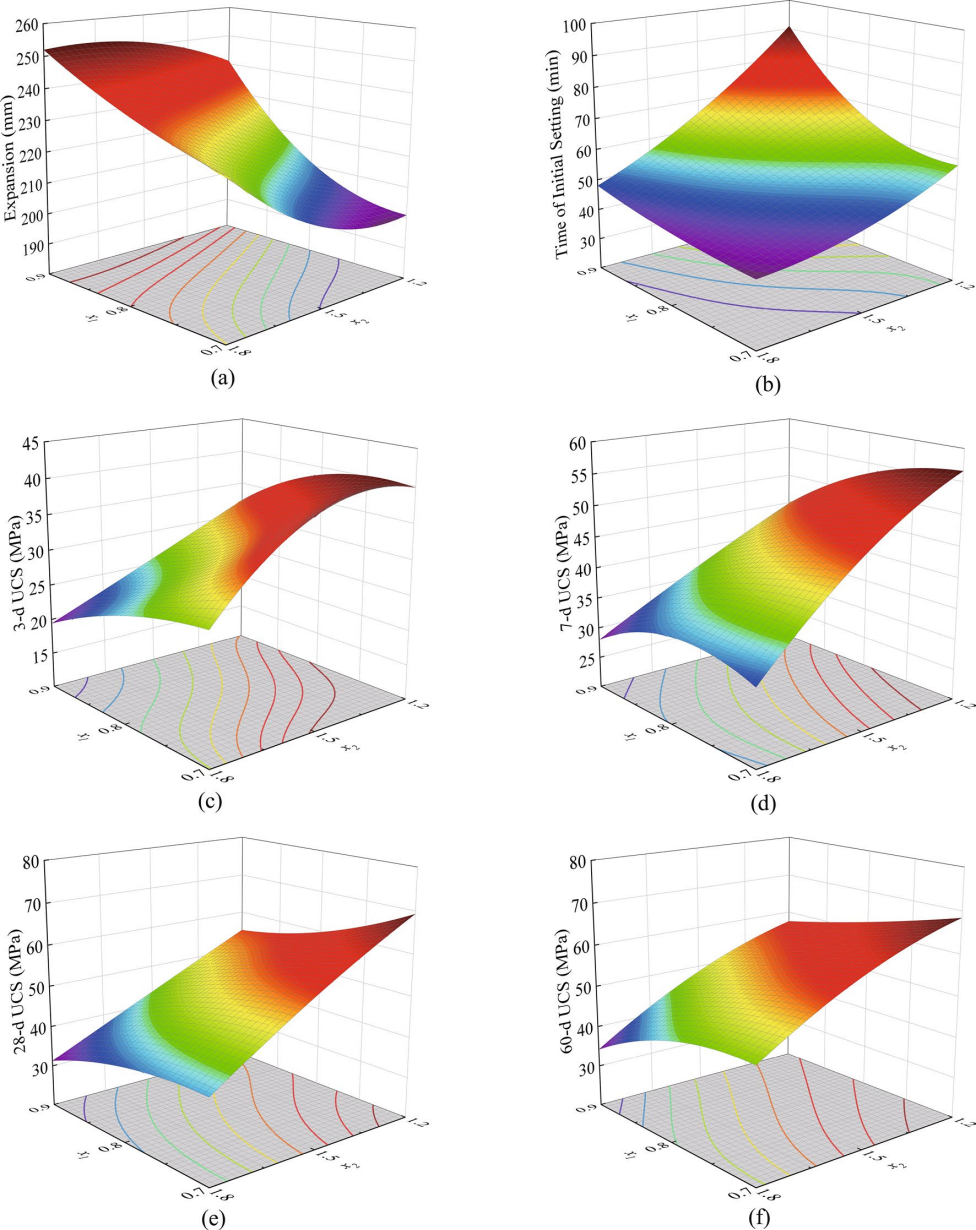
3D response surface diagrams for the effects of L/S and Ms on (**a**) Fluidity; (**b**) Initial setting time; (**c**) 3-d UCS; (**d**) 7-d UCS; (**e**) 28-d UCS; and (**f**) 60-d UCS.

According to the regression models, as shown in Fig. 7, the 3D response surface diagrams of different response values could be obtained. The response values were displayed from purple to red in order of smallest to largest. The contours projected from the response surface to the bottom could be used to reflect the change in the response value, and the denser the contours were, the faster the response values changed, then the greater the influence of the factors were. It can be seen from Fig. 7 that the interactions of factors in the design interval were weak, and the maximum value point did not appear in the single response surface. There was a constraint relationship between the response values. For example, the increase of L/S had a positive effect on the fluidity, but it prolonged the setting time and reduced the mechanical properties. The reduction of Ms was beneficial to the mechanical properties, but it affected the workability of the paste and made it difficult to stir and form. These showed that the influence of L/S and Ms on the fluidity, setting time, and compressive strength in the selected interval needed to be considered comprehensively, not for one certain response value.

### Optimum mix of MSGP based on CCD method

Just as mentioned above, for anisotropic concrete materials, a single response value was not an optimization goal of its performance, but should comprehensively considered the workability, mechanical property and other performances. According to the actual condition, the target fluidity is 220 mm, and the initial setting time is between 30 and 50 min considering the quick setting of GGBFS, then the optimum mix ratio based on the maximum 28-d UCS is: L/S = 0.75, Ms = 1.55. Under the same test conditions, the MSGP was produced with the optimum mix ratio. The measured fluidity was 216 mm, the initial setting time was 53 min, and the 28-d UCS was 53.10 MPa. Table 8 compares the experimental and predicted values of the optimum mix ratio. The mean absolute percentage error (MAPE) of the experimental and predicted values was calculated according to the following formula^67^.

7 $$ {\text{MAPE }}\left( {\text{\% }} \right){ = }\frac{{\text{Experimental - Predicted}}}{{{\text{Experimental}}}} \times 100\% $$

**Table 8:** Comparison between the experimental and predicted values of the optimized mix.

Term	Units	Experimental	Predicted	MAPE (%)
Fluidity	mm	216	220	− 1.81
Initial setting time	min	53	50	6.00
28-d UCS	MPa	53.10	51.36	3.28

### Microstructural analysis

In order to further study the effects of L/S and Ms on the polymerization reaction, workability, and mechanical properties of MSGP, the microstructure was determined by XRD-SEM method. It was explained from two aspects: hydration reaction products and micropore changes.

### XRD

**Figure 8:**
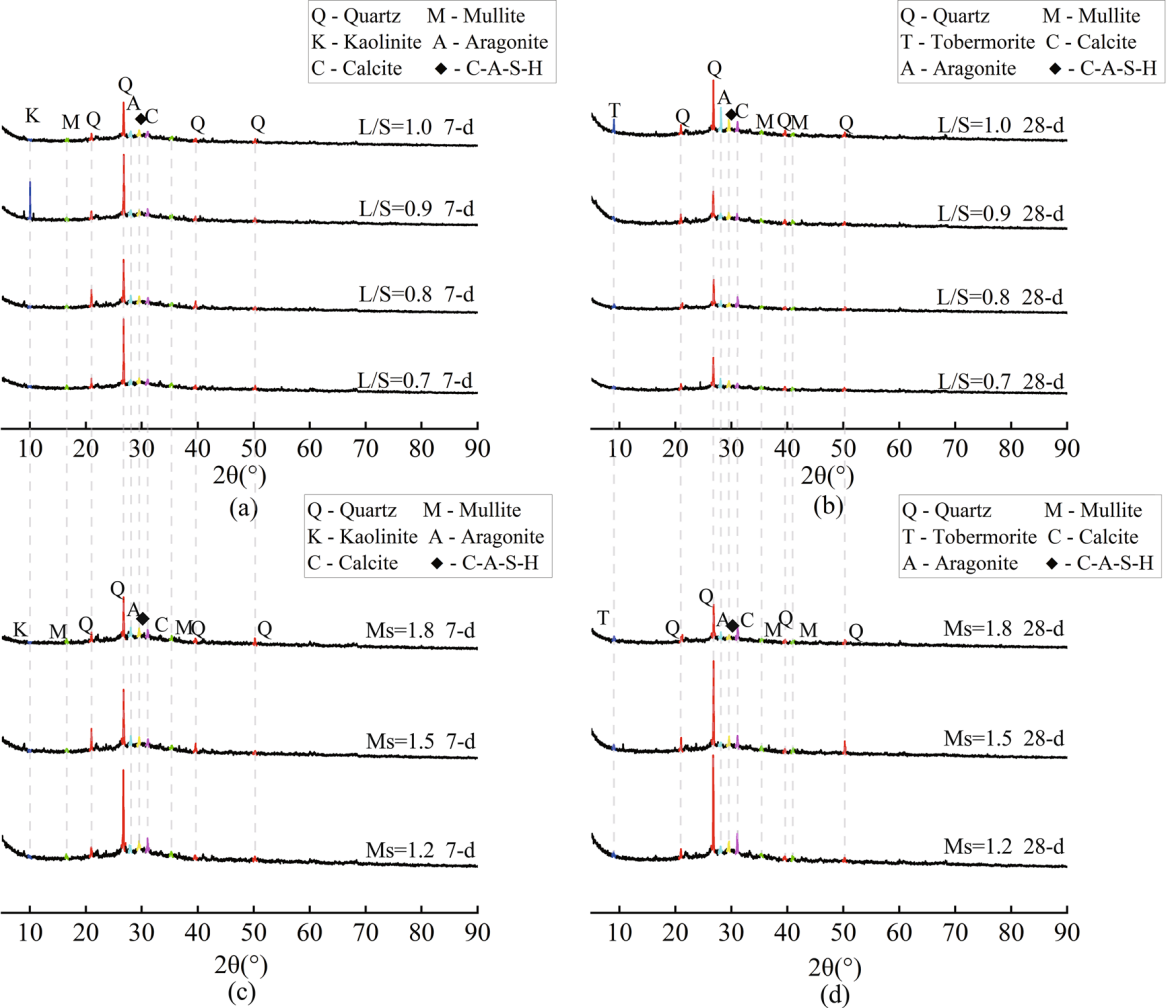
XRD patterns of MSGP: (**a**) Ms = 1.5, 7-d; (**b**) Ms = 1.5, 28-d; (**c**) L/S = 0.8, 7-d; (**d**) L/S = 0.8, 28-d.

Figure 8 shows the XRD patterns of MSGP after curing for 7 and 28 days. In the initial hydration, the formation of quartz (SiO_2_), mullite (3Al_2_O_3_·2SiO_2_), kaolinite (Al_2_Si_2_O_5_(OH)_4_), calcite (CaCO_3_), etc., as well as the diffuse peak of C–A–S–H (around 2θ = 30°), could be observed. The phase of kaolinite is attributed to the unreacted metakaolin^68^. The presence of calcite is due to the fact that, ambient CO_2_ reacted with during the polymerization reaction^69^. The Ca(OH)_2_ is generated by the reaction between Ca^2+^ dissolved in the MSGP samples and OH^−^ in the alkali solution. As hydration continued, the tobermorite ((CaO)_x_–SiO_2_–zH_2_O) began to be observed in the spectrum^70^. At the same time, it was found that the formation of C–A–S–H gel and its nearby aragonite and calcite increased. The dissolved alumina in the precursors react with OH^−^ in the alkali solution and form tetrahedral [H_3_AlO_4_]^−^ and octahedral [Al(OH)_6_]^3−^. Then [H_3_AlO_4_]^−^ further reacts with Ca^2+^ to form C–A–S–H gel^49,71,72^. L/S and Ms did not affect the phase of the hydration products too much, and then they had little effect on the final hydration products. However, it promoted the polymerization reaction of the paste because the dissolution rate of the aluminosilicate precursor changed.

### SEM

**Figure 9:**
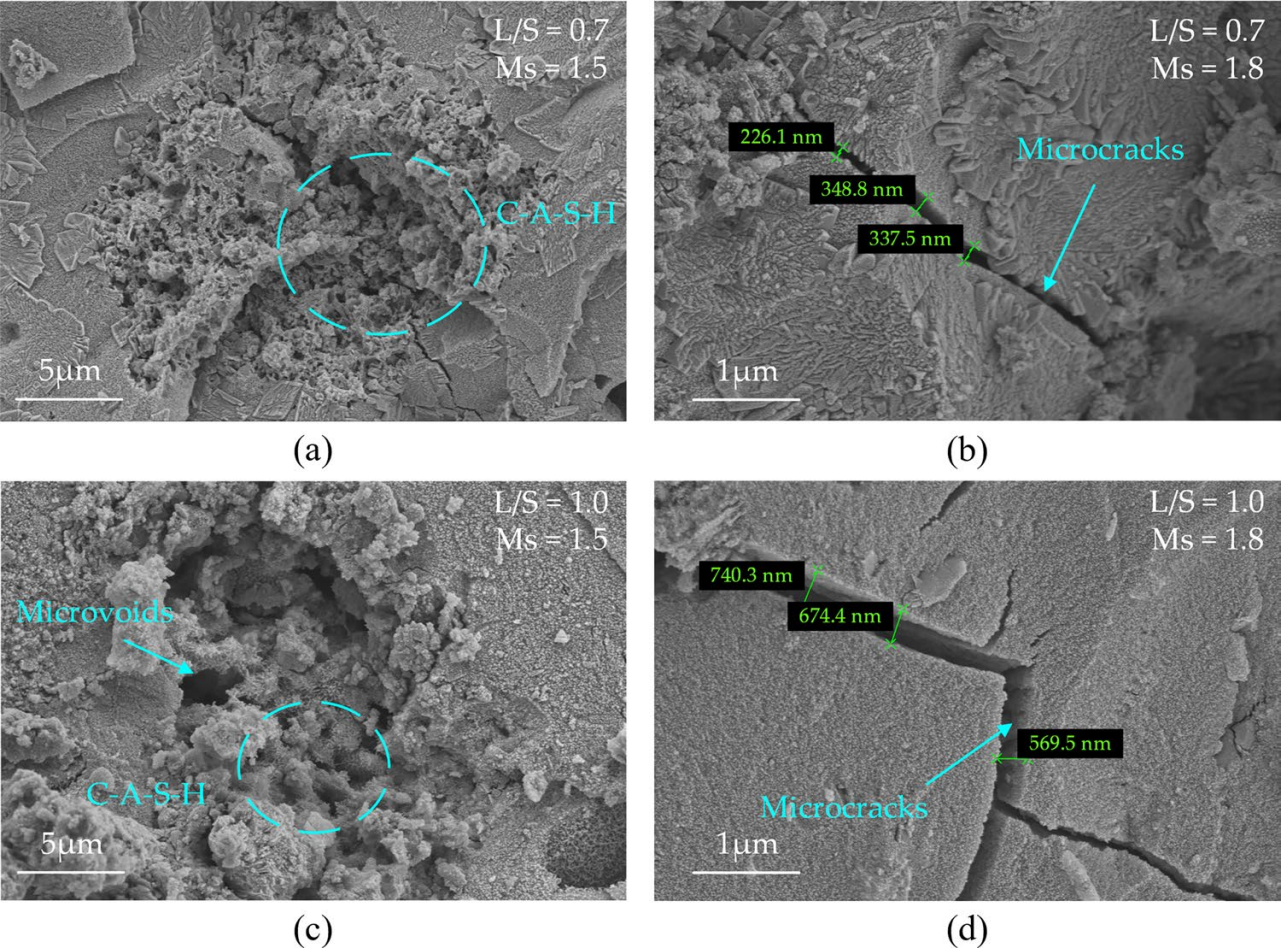
28-d SEM diagram of four mix proportion: (**a**) L/S = 0.7, Ms = 1.5; (**b**) L/S = 0.7, Ms = 1.8; (**c**) L/S = 1.0, Ms = 1.5; (**d**) L/S = 1.0, Ms = 1.8.

The SEM diagrams of 28-d MSGP are shown in Fig. 9. It could be seen from the diagram that for MSGP with constant Ms, the increase of L/S had an adverse effect on the compactness of the gel structure. It was reflected in the microstructure with the rough and porous gel morphology and the further increase of the width of the microcracks. The mechanical properties of MSGP worsened due to the presence of pores and cracks ^69^. This is consistent with the above results of experiments. On the other hand, the increase of Ms improved the workability of MSGP. The frictional resistance between the particles was reduced due to the action of the sodium silicate micelle. However, the rapid polymerization reaction was not conducive to the binding of low active ingredients, which appeared porous and disordered from a micro view, then reducing the macroscopic mechanical properties.

## Conclusions

In this research, the impacts of liquid–solid ratio (L/S) and modulus of sodium silicate (Ms) on the workability and mechanical performances of MK-GGBFS based geopolymer paste (MSGP) were investigated. Then, the optimum mix ratio was found using the central composite design method for all the three properties simultaneously. The main conclusions are listed below:

1. The synergy between metakaolin (MK) and ground granulated blast furnace slag (GGBFS) is good. The setting time can be extended effectively by partially replacing GGBFS with MK, overcoming the defect of quick harden of GGBFS-based geopolymer.
2. When L/S was raised from 0.7 to 1, the workability was effectively improved. When Ms was 1.5, the fluidity increased from 209 to 273 mm, and the initial setting time prolonged from 46 to 71 min. With the increasement of Ms from 1.2 to 1.8, the fluidity increased from 201 to 227 mm when L/S was 0.7, but the initial setting time shortened slightly from 58 to 41 min.
3. The regression models established by central composite design method fitted well on the six response values, and the R^2^ were all above 0.98. The optimum mix ratio with L/S ratio of 0.75 and Ms value of 1.55 was obtained. The measured fluid is 216 mm, the initial setting time is 53 min, and the 28-d unconfined compressive strength is 53.1 MPa.

## Data Availability

All data generated or analyzed during this study are included in this published article.
